# Blood-based proteomic signatures of spontaneous menopause: Implications for later-life brain aging and Alzheimer’s disease risk

**DOI:** 10.21203/rs.3.rs-8752767/v1

**Published:** 2026-02-12

**Authors:** Madeline Wood Alexander, Jennifer S. Rabin, Michelle Caunca, Allesandra Iadipaolo, Louisa Cornelis, Nina Miolane, Albert Pham, Julia Borger, Valentina Diaz, Emily W. Paolillo, Joel Kramer, Laura Pritschet, Caitlin Taylor, Matthew S. Panizzon, Ramiro Eduardo Rea Reyes, Marisa N. Denkinger, Nicholas J. Ashton, Sterling C. Johnson, Emily G. Jacobs, Rowan Saloner, Kaitlin B. Casaletto

**Affiliations:** 1 Hurvitz Brain Sciences Program, Sunnybrook Research Institute, Toronto, Ontario, Canada, M4N 3M5.; 2 Rehabilitation Sciences Institute, Temerty Faculty of Medicine, University of Toronto, Toronto, Ontario, Canada, M5G 1V7.; 3 Harquail Centre for Neuromodulation, Sunnybrook Health Sciences Centre, University of Toronto, Toronto, Ontario, Canada, M4N 3M5.; 4 Division of Neurology, Department of Medicine, Sunnybrook Health Sciences Centre, University of Toronto, Toronto, Ontario, Canada, M4N 3M5.; 5 Neurovascular Division, Department of Neurology, Weill Institute for Neurosciences, University of California, San Francisco, California, USA, 94158.; 6 Department of Psychological & Brain Sciences, University of California, Santa Barbara, Santa Barbara, California, USA, 93106.; 7 Ann S. Bowers Women’s Brain Health Initiative, University of California, California, USA, 93106.; 8 Neuroscience Research Institute, University of California, Santa Barbara, Santa Barbara, California, USA, 93106.; 9 Department of Physics, University of California, Broida Hall, Santa Barbara, CA, USA, 93106.; 10 Edward and Pearl Fein Memory and Aging Center, Department of Neurology, Weill Institute for Neurosciences, University of California, San Francisco, California, USA, 94158.; 11 Department of Psychiatry, University of Pennsylvania, Philadelphia, PA, USA, 19104.; 12 Center for Behavior Genetics of Aging, School of Medicine, University of California, San Diego, La Jolla, CA, USA, 92093.; 13 Wisconsin Alzheimer’s Disease Research Center, School of Medicine and Public Health, University of Wisconsin, Madison, WI, USA, 53792-2420.; 14 Banner Sun Health Research Institute, Sun City, AZ, USA, 85351.; 15 Banner Alzheimer’s Institute, Phoenix, AZ, USA, 85006.; 16 Wisconsin Alzheimer’s Institute, School of Medicine and Public Health, University of Wisconsin, Madison, WI, USA, 63726.

## Abstract

Menopause is a hallmark process in biological aging that has been associated with later life neurodegenerative risk. We leveraged proteomics data from multiple cohorts (*N*>3,000) to identify biological changes underlying menopause and its links to brain aging. In *N*=80 rigorously-phenotyped pre-, peri-, and postmenopausal women with serum NULISAseq proteomics, spontaneous menopause was characterized by dysregulation in inflammatory, synaptic, metabolic, and Alzheimer’s disease (AD) biologic processes, which tracked with hormones and not age. Pro-inflammatory protein upregulation was especially pronounced in women with vasomotor symptoms. In two cohorts of older women (*N*=94; *N*=100), menopause-related proteomic elevations associated with poorer cognitive outcomes and plasma AD biomarkers. Finally, validation analyses in age-matched pre- and postmenopausal women with plasma Olink proteomics (*N*=2,814) replicated the observed proteomic shifts and revealed menopause-related upregulation of additional inflammatory and hormone signaling processes. The molecular signatures of menopause may inform biomarkers or therapeutic targets for brain health in women.

## INTRODUCTION

Accumulating data point towards menopause as a critical inflection point in the aging process.^1^ Universal to all midlife individuals with ovaries, menopause is characterized by a steep decline in ovarian hormones, chiefly 17β-estradiol and progesterone, along with a sustained rise in follicle-stimulating hormone (FSH). These endocrine shifts are associated with widespread multi-system health effects, including altered immune function, increased cardiovascular and metabolic risk, reduced synaptic plasticity in the brain, and accelerated epigenetic aging.^1–4^

Menopause precipitates increases in risk for several age-related chronic diseases,^3,5^ with particularly strong links to brain aging.^6–10^ Menopause-induced changes to the neuroendocrine, neuroimmune, and cerebrovascular environments may contribute to the two-fold greater lifetime risk for Alzheimer’s disease (AD) in women vs. men.^11^ Supporting this, neuroimaging studies have demonstrated increases in Aβ, tau, hypometabolism, and atrophy in AD-vulnerable brain regions in peri- and postmenopausal women compared to premenopausal women and age-matched men.^12–15^ Furthermore, vasomotor symptoms of menopause (i.e., hot flashes, night sweats) have been associated with poorer memory performance, white matter hyperintensities, and worse levels of plasma Aβ.^16–18^ Together, these data position menopause as a neurologic transition with potential relevance for understanding brain aging and AD.

Despite these emerging links, human research investigating the biological pathways by which menopause influences brain aging is scarce. Technological advances in the characterization of the human proteome from blood now enable interrogation of molecular signatures underlying ovarian aging, neurodegeneration, and dementia risk in living humans. The limited existing proteomics studies of menopause have identified changes in targets involved in metabolic, vascular, and immune processes.^19,20^ However, existing studies did not employ rigorous definitions of menopause or proteomic platforms targeted at brain aging and neurodegenerative biomarkers. Further, no studies have linked the proteomic profiles of menopause to cognitive or brain health outcomes.

We addressed these gaps by characterizing a blood-based proteomic signature of spontaneous menopause in a cohort of midlife women with a restricted age range (43–58) and rigorous menopause staging.^21^ We employed ultra-sensitive NUcleic acid Linked Immuno-Sandwich Assay (NULISAseq) technology to quantify over 120 proteins implicated in brain aging and neurodegenerative disease.^22^ We identified proteomic changes associated with the menopause transition and evaluated their relationships to hormones and menopause symptoms. To probe the relevance of menopause biology to later-life brain aging, we examined whether menopause-associated proteins were related to cognitive outcomes and plasma AD biomarkers in two independent cohorts of older women. Finally, we validated menopause-related proteomic changes and identified additional menopause pathways in a large independent cohort of age-matched pre- and postmenopausal women with an orthogonal proteomics platform.

## METHODS

### Spontaneous menopause cohort participants

We recruited approximately equal numbers of pre-, peri-, and postmenopausal cisgender women (*N*=80) plus age-matched cisgender men (*N*=36) to participate in the UCSB Healthy Aging Study (HAS). Menopause was staged following STRAW+10 criteria.^21^ Participants were aged 35–60, not currently taking menopausal hormone therapy, and did not have any major medical conditions. Participants who were not taking hormonal contraceptives were prioritized for recruitment, resulting in a sample that was largely free of exogenous hormones (*N*=1 on oral contraceptives). Female participants were required to have an intact uterus and no history of bilateral oophorectomy. Female participants self-reported how often they experienced menopause symptoms including hot flashes, night sweats, vaginal dryness, and irritability in the last two weeks, on a scale of “not at all”, “1–5 days”, “6–8 days”, “9–13 days”, or “every day”. Symptoms were dichotomized as present versus absent.

### Endocrine assays

Serum from a venous blood draw was analyzed for gonadotropin and sex steroid hormones: FSH, 17β-estradiol, progesterone, sex-hormone binding globulin (SHBG), dehydroepiandrosterone sulfate (DHEAS), and testosterone (Extended Data).

### Blood proteomics

Serum (menopause cohort) or plasma (aging cohorts, see below) was analyzed on the NULISAseq CNS panel (~120 proteins) using the automated Alamar Argo HT workflow (Extended Data).

### Aging cohorts

To evaluate the relevance of menopause-related biological changes to later-life brain aging and AD risk, we used plasma NULISAseq proteomics, cognitive, and orthogonal AD biomarker data from women in two independent aging cohorts.

#### Wisconsin Registry for Alzheimer’s Prevention (WRAP)

WRAP is an observational study enriched for participants with a parental history of AD. Participants completed clinical/cognitive testing and blood collection approximately every two years. Female participants self-reported histories of hormone therapy use, oophorectomy, and menopause symptoms (hot flashes, night sweats, sexual dysfunction, problems sleeping, mood swings, depression, plus total symptom count). Composite cognitive scores were calculated by averaging sample-based z-scores on tests for memory and executive function (Extended Data). Plasma p-tau217/Aβ42 was measured on Lumipulse G (Fujirebio). Amyloid positivity was determined using a threshold of >0.008.^23^ Plasma samples from a subset of recent WRAP visits (2017–2024) were analyzed for 122 proteins on the NULISAseq CNS kit.^24^ Given the lack of sufficient follow-up after the NULISAseq blood draw, we restricted analyses to cross-sectional data. Our sample included female participants who had either cognitive (*N*=93) or orthogonal plasma AD biomarker data (*N*=91) available within 30 days of NULISA blood collection (total *N*=94).

#### UCSF Brain Aging Network for Cognitive Health (BrANCH)

BrANCH is a study of community-dwelling, functionally intact participants who undergo annual neuropsychological testing and blood draws. Female participants self-reported hormone therapy use and oophorectomy. Domain-specific cognitive scores were computed by averaging sample-based z-scores for memory and executive function (Extended Data). Baseline plasma p-tau181/Aβ42 was assayed on Simoa (Quanterix). Amyloid positivity was determined using a sample-specific, batch-harmonized p-tau181/Aβ42 threshold (Extended Data). Baseline plasma was also analyzed for 131 proteins on the NULISAseq CNS platform. Our analytic sample included *N*=100 female participants with NULISAseq who had either cognitive (*N*=100) or orthogonal plasma AD biomarker data available (*N*=60). We examined longitudinal cognitive decline and cross-sectional associations with plasma AD biomarkers given the limited follow-up data for the latter.

### Cross-cohort, cross-platform validation in UK Biobank

To evaluate whether spontaneous menopause proteomic shifts were robust to cohort and proteomic platform, we compared menopause proteins detected in the midlife menopause cohort on NULISAseq with proximity extension assay (PEA) Olink plasma proteomic data^25^ in a large cohort of 2,814 women from UK Biobank (Extended Data). Premenopausal (*N*=1,407) and postmenopausal (*N*=1,407) women were propensity matched on age within a range of 45–60 years, comparable to the UCSB HAS cohort. Menopause status was self-reported as pre- or postmenopausal.

### Analyses

Analyses were conducted in R (v4.5.0). Descriptive statistics and ANOVA, t-tests, and χ^2^ tests summarized demographic and clinical characteristics in each cohort.

### Spontaneous menopause cohort

#### Identifying menopause proteins.

Age-adjusted analyses of covariance (ANCOVA) compared levels of 126 NULISA proteins by menopause stage (i.e., pre/peri/post). Menopause proteins were identified as those that differed between pre- and postmenopausal women per nominal statistical significance (i.e., unadjusted *p*<.05). All further analyses focused exclusively on these proteins. To disentangle effects of menopause stage versus chronological age, we examined partial correlations between each of the identified menopause proteins and age in women (adjusting for menopause status), and between each protein and age in men.

#### Computing a menopause signature score.

To reduce data dimensionality and summarize menopause-related proteomic changes, we performed a principal components analysis on the identified menopause proteins in all midlife women, and extracted the first component (i.e., PC1) as a “menopause proteomic signature.” To limit comparisons, the menopause signature was used as the primary measure for all further analyses, with effects of individual proteins probed in post hoc analyses. Separate age-adjusted regressions tested the associations of menopause stage and sex with the menopause signature.

#### Evaluating associations of hormones and menopause symptoms with the menopause signature score.

While menopause definitionally involves endocrine shifts, different hormones may relate to unique aspects of menopause-related biological changes. To probe this, we used age-adjusted regressions to test associations between sex hormones and the menopause signature in all midlife women. Among peri- and post-menopausal women, separate regressions tested associations of menopause symptoms with the menopause signature, adjusting for age and menopause stage.

### Aging cohorts

We extracted loadings and summary statistics for the menopause signature (i.e., PC1) calculated in the spontaneous menopause cohort to compute an equivalent score in each aging cohort (Extended Data).

#### Associations between menopause symptoms and the menopause proteomic signature in later life.

In WRAP, separate regressions tested the associations of history of menopause symptoms with the menopause signature, adjusting for age, history of hormone therapy, and bilateral oophorectomy.

#### Examining relevance of the menopause proteomic signature to AD risk.

In WRAP, separate regressions tested associations of the menopause signature with memory, executive function, and plasma p-tau217/Aβ42. Cognitive models adjusted for age and education; the AD biomarker model adjusted for age and body mass index (BMI). In BrANCH, separate linear mixed-effects models with random slopes and intercepts tested the interaction between baseline menopause signature score and time on memory and executive function, adjusting for baseline age and education. A regression tested the association between the menopause signature with plasma p-tau181/Aβ42, adjusting for age and BMI. P-tau/Aβ42 ratios were expressed in sample-based z-scores for cross-cohort interpretability.

### Cross-cohort, cross-platform validation

In the age-matched sample of pre- and postmenopausal women in the UKB Biobank, age-adjusted regression models tested associations between menopause status (post/pre) and levels of 2,923 Olink proteins, with false-discovery rate (FDR) correction. Using these differential abundance results, we performed gene ontology (GO) enrichment analyses using the publicly-available GOparallel code (https://www.github.com/edammer/GOparallel).^26^ Finally, for the menopause proteins identified in UCSB (NULISAseq) that were measured on Olink, we examined whether these proteins also differed between pre- and postmenopausal women in the UKB, including estimated proteomic effect sizes between cohorts.

All studies were approved by research ethics boards and all participants provided informed consent.

## RESULTS

### Spontaneous menopause cohort

**Figure 1.**
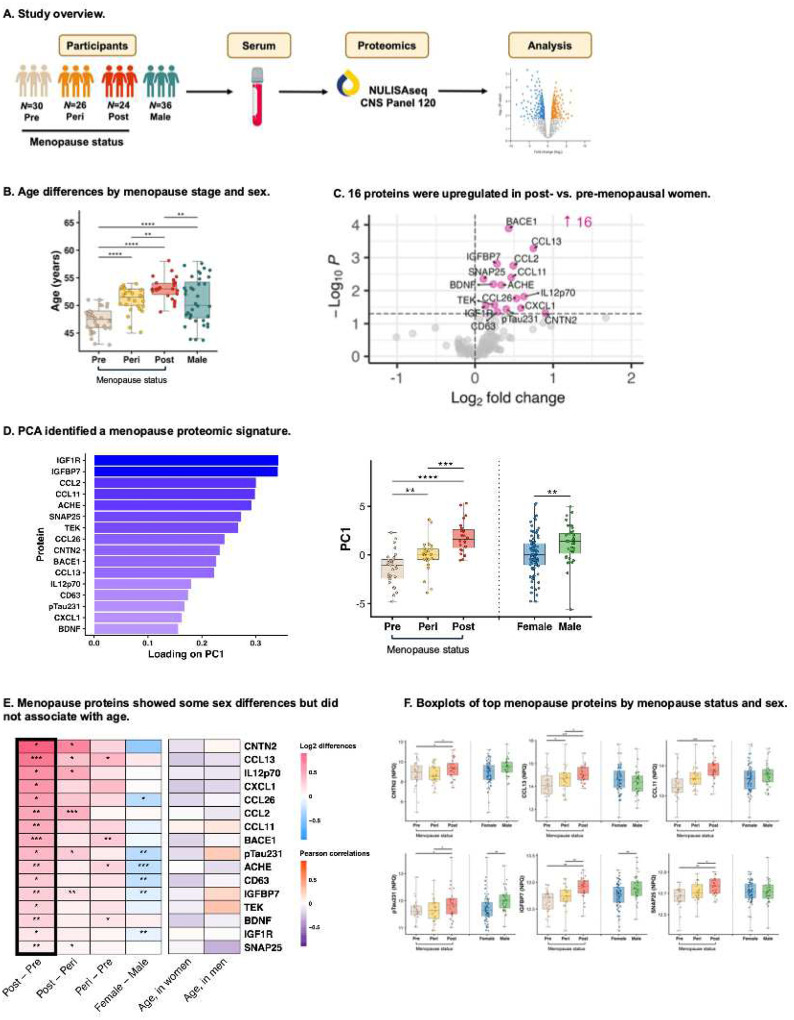
Blood-based proteomic signature of spontaneous menopause. **A.** Blood was collected in 30 premenopausal, 26 perimenopausal, and 24 postmenopausal women, plus 36 men for comparison. Serum was analyzed for 127 proteins via NUcleic acid Linked Immuno-Sandwich Assay (NULISAseq CNS panel). Age-adjusted analyses identified differences in protein levels across menopause stages and sex. **B.** Participant ages ranged from 43 to 58 years. Despite significant group differences in expected directions, menopause stages exhibited substantial overlap in age. **C.** Analysis of variance (ANOVA) revealed 16 proteins that were significantly upregulated in postmenopausal women relative to premenopausal women, reflecting immune, synaptic, metabolic, and neurodegenerative biology. **D.** Principal components analysis (PCA) performed on these 16 proteins identified a menopause signature score (i.e., PC1) that increased across menopause stages. **E.** In age-adjusted analyses, many of the 16 menopause proteins showed significant elevations across menopause stages (i.e., pre-, to peri-, to post), and some showed sex differences, but none significantly correlated to age in women (after accounting for menopause status) or to age in men. **F.** Boxplots of top menopause proteins revealed stepwise increases across the menopause transition. *p<.05, **p<.01, ***p<.001.

We included *N*=30 premenopausal, *N*=26 perimenopausal, and *N*=24 postmenopausal women, plus *N*=36 men for comparison (Table S1; Fig.1A). In women, age ranged from 43–58, and age differences by menopause stage were statistically significant but small (Fig.1B). The vast majority were late premenopausal (i.e., STRAW stages −3b, −3a), perimenopausal (i.e., −2, −1), or early postmenopausal (i.e., 1a, 1b, 1c) (*N*=1 postmenopausal woman stage 2; *N*=1 perimenopausal woman for whom exact stage could not be ascertained). There were no significant age differences by sex and no menopause group differences in BMI. Where not included in the text, statistical output for main analyses is presented in Extended Data Tables S4-S13.

#### Inflammatory, synaptic, and neuropathology-related proteins were elevated in postmenopausal women.

We first examined differential serum protein abundance in postmenopausal vs. premenopausal women (Fig.1C). 16 proteins were elevated in postmenopausal women, reflecting inflammatory (CCL13, IL-12p70, CXCL1, CCL26, CCL2, CCL11, TEK, CD63), synaptic/neuronal network (CNTN2, ACHE, SNAP-25, BDNF), metabolic (IGFBP7, IGF1R), and AD (BACE1, p-tau231) biology. We performed PCA on these 16 targets to estimate a “menopause proteomic signature” composite score. The first principal component (PC1) explained 28.7% of the variance in menopause proteins (Fig.S2), whereby higher values reflected a more postmenopausal-like proteome (Fig.1D). Confirming this, age-adjusted analyses revealed stepwise increases in menopause signature scores across the menopause transition (post [vs. peri]: β=2.20, 95% CI=1.178, 3.21, *p*<.0001, peri [vs. pre]: β=1.87, 95% CI=0.772, 2.98, *p*=.001). We assessed stability of PC1 loadings using bootstrap resampling (1000 iterations), which showed low *SD*s (<0.08) and high sign consistency (>0.95) across all proteins (Fig.S3).

Age was not associated with PC1 scores after adjusting for menopause stage, suggesting that proteomic changes were not driven by chronological age (β= −0.14, 95% CI= −0.31, 0.03, *p*=.11). We computed the same composite score in men to facilitate sex comparisons. Midlife men had higher PC1 scores than midlife women overall adjusting for age (β=1.13, 95% CI=0.27, 1.90, *p*=.009), possibly reflecting the shift to relatively greater androgen dominance that women undergo during menopause. In men, age did not correlate to PC1 scores (cor=−0.02, *p*=.89).

Notably, after accounting for age, p-tau231 was the only canonical AD biomarker (versus p-tau181, ptau217, AB40, AB42, GFAP, NFL) that significantly differed by menopause stage (Fig.S4).

#### Menopause proteins tracked with estradiol and FSH.

**Figure 2.**
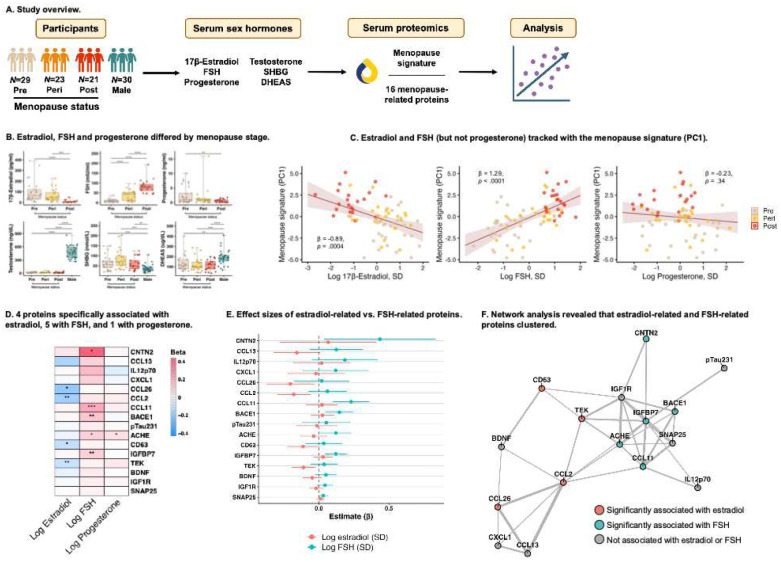
Menopause proteins tracked with estradiol and follicle-stimulating hormone (FSH). **A.** Serum was analyzed for sex hormones: 17β-estradiol, follicle-stimulating hormone (FSH), and progesterone (in women only), plus testosterone, sex-hormone binding globulin (SHBG), and dehydroepiandrosterone sulfate (DHEAS) in all participants. Analyses examined associations between menopause proteins and hormone levels. **B.** Estradiol, FSH, and progesterone differed by menopause stage. **C.** Age-adjusted regression models revealed that estradiol and FSH (but not progesterone) significantly related to the menopause signature score. **D.** In models simultaneously testing associations of hormone levels with individual proteins, FSH was uniquely associated with 5 proteins, estradiol was uniquely associated with 4, and progesterone was uniquely associated with 1. **E.** A forest plot of beta coefficients for estradiol and FSH from age- and progesterone-adjusted analyses showed that menopause proteins demonstrate specificity in their associations with sex hormones. CNTN2, CCL11, BACE1, ACHE, and IGFBP7 are predicted only by FSH, and CCL26, CCL2, CD63, and TEK only by estradiol. **F.** Network analysis demonstrated that estradiol-associated (CCL13, CCL26, CCL2, TEK) and FSH-related (CNTN2, CCL11, BACE1, ACHE, IGFBP7) proteins clustered in two groups. *p<.05, **p<.01, ***p<.001.

Estradiol, FSH, and progesterone significantly differed by menopause stage, while testosterone, SHBG, and DHEAS did not (Table S1, Figs.2A–B). Therefore, we focused on the former three hormones in proteomic analyses. Age-adjusted models showed the menopause proteomic signature inversely associated with estradiol (β= −0.89, 95% CI= −1.37, −0.41, *p*=.0004), positively associated with FSH (β=1.29, 95% CI=0.78, 1.80, *p*<.0001), and did not associate with progesterone (β= −0.23, 95% CI= −0.71, 0.25, *p*=.34) (Fig.2C). When simultaneously modeling all three hormones together, only FSH was significantly associated with the menopause signature (FSH: β=1.13, 95% CI=0.51, 1.78, *p*=.0005; estradiol: β= −0.43, 95% CI= −0.97, 0.11, *p*=.12; progesterone: β=0.24, 95% CI= −0.20, 0.68, *p*=.28). Post-hoc analyses examining individual proteins showed distinct hormone-protein relationships such that CCL26, CCL2, CD63, and TEK inversely tracked with 17β-estradiol levels; CNTN2, CCL11, BACE1, ACHE, and IGFBP7 positively tracked with FSH; and only ACHE tracked with progesterone (Figs.2D–E). A network analysis estimating pairwise correlations at a threshold of r=0.3 illustrated that estradiol-related proteins and FSH-related proteins were generally clustered in two groups (Fig.2F).

#### Women with vasomotor symptoms showed the most pronounced increases in inflammatory menopause proteins.

**Figure 3.**
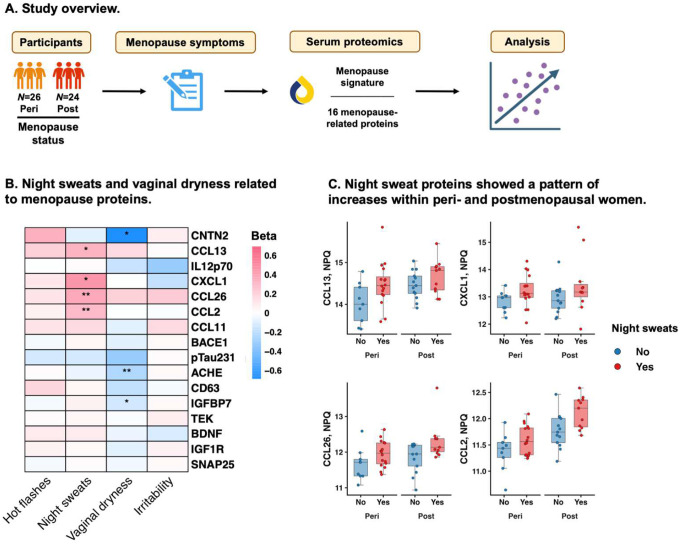
Menopause symptoms associated with menopause protein levels in peri- and postmenopausal women. **A.** Peri- and postmenopausal women self-reported hot flashes, night sweats, vaginal dryness, and irritability. Analyses tested associations of menopause symptoms with menopause signature scores and individual proteins, adjusted for age and menopause stage. **B.** Women with night sweats had significantly elevated levels of CCL13, CXCL1, CCL26, and CCL2. Conversely, women with vaginal dryness had significantly lower levels of CNTN2, ACHE, and IGFBP7. **C.** Boxplots of top night sweats proteins showed that women with night sweats had a pattern of elevated protein levels within each menopause stage.

In peri- and postmenopausal women, we tested whether menopause symptoms were associated with the menopause proteomic signature (Fig.3A). Night sweats were related to higher (i.e., more postmenopausal) scores on the menopause signature (β=0.94, 95% CI=0.00, 1.88, *p*=.05), while vaginal dryness was associated with lower scores (β= −1.15, 95% CI= −2.17, −0.13, *p*=.03). There were no associations between hot flashes or irritability and the menopause signature (hot flashes: β=0.43, 95% CI= −0.55, 1.42, *p*=.38; irritability: β=0.32, 95% CI= −0.74, 1.38, *p*=.55). In post hoc analyses, CXCL1, CCL26, CCL13, and CCL2 were significantly upregulated in women with night sweats, while CNTN2, ACHE, and IGFBP7 were downregulated in women with vaginal dryness (Figs.3B–C).

### Aging cohorts

#### Menopause symptoms were associated with menopause protein levels in later life.

**Figure 4.**
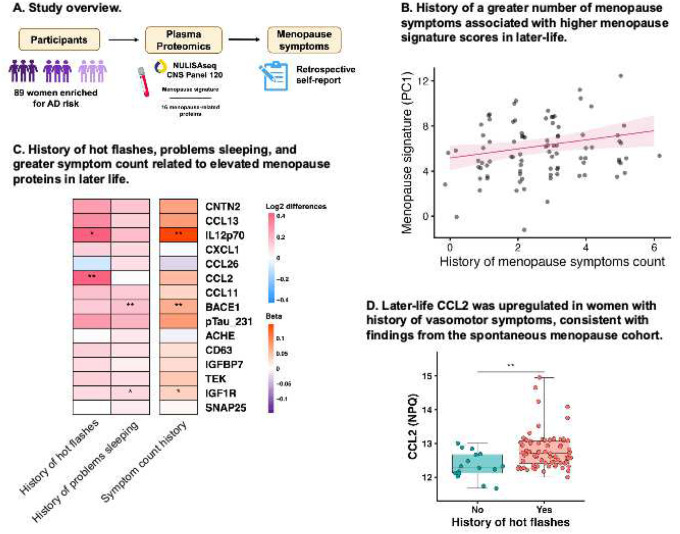
History of menopause symptoms relates to menopause protein levels years to decades later. **A.** In aging cohort 1 (WRAP), women self-reported their history of menopause symptoms, including hot flashes, night sweats, sexual dysfunction, problems sleeping, mood swings, and depression. Analyses evaluated associations of menopause symptom history with menopause signature scores and individual proteins, adjusted for age, history of hormone therapy, and bilateral oophorectomy. **B.** History of greater total menopause symptom count was associated with higher menopause signature scores in analyses adjusted for age, history of hormone therapy, and bilateral oophorectomy. **C.** Among symptoms that significantly associated with menopause signature scores (i.e., hot flashes, problems sleeping, and total symptom count), post hoc analyses tested associations with individual menopause proteins, adjusted for age, history of hormone therapy, and bilateral oophorectomy. History of hot flashes associated with increased IL12p70 and CCL2, problems sleeping with increased BACE1 and IGF1R, and symptom count with increased IL12p70, BACE1, and IGF1R. **D.** A boxplot visualization of CCL2 level by history of hot flashes showed higher levels in women with hot flashes vs. without. *p<.05, **p<.01, ***p<.001.

In WRAP (Table S2), we examined whether history of menopause symptoms showed associations with the identified menopause proteins years to decades after menopause (Fig.4A). Higher menopause signature scores were associated with hot flashes (β=1.45, 95% CI=0.14, 2.76, *p*=.03), problems sleeping during menopause (β=1.11, 95% CI=0.08, 2.15, *p*=.04), and total menopausal symptom count (β=0.40, 95% CI=0.04, 0.76, p=.03; Fig.4B). Associations between other menopause symptoms and the menopause signature were not significant (mood swings: β=1.04, 95% CI= −0.03, 2.11, *p*=.06; night sweats: β=0.10, 95% CI = −0.93, 1.13, *p*=.84; depression: β=0.45, 95% CI= −0.86, 1.77, *p*=.50; sexual dysfunction: β=1.01, 95% CI= −0.57, 2.61, *p*=.21). Post-hoc analyses showed that hot flashes associated with increases in IL12p70 and CCL2; problems sleeping with increases in BACE1 and IGF1R; and total symptom count with increases in IL12p70, BACE1, and IGF1R (Fig.4C). CCL2 was the only individual protein associated with vasomotor symptoms in both older women and midlife women, suggesting a link between vasomotor symptoms and CCL2 that may persist into later life (Fig.4D).

#### Menopause proteomic signatures related to markers of adverse brain aging and AD risk in later life.

**Figure 5.**
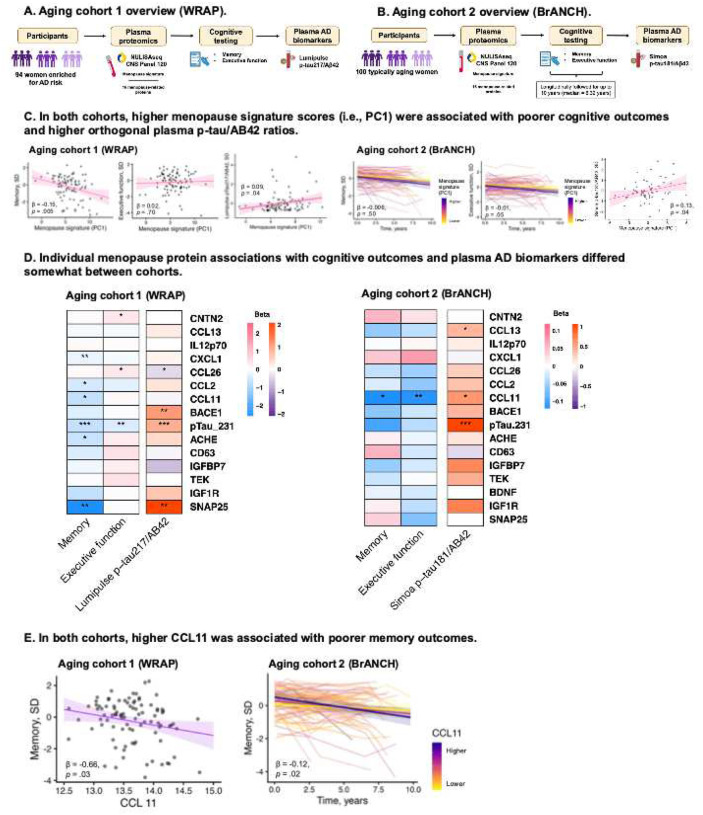
In later life, elevated menopause proteomic signatures associated with poorer cognitive outcomes and higher levels of plasma AD biomarkers. In aging cohorts 1 and 2 (WRAP (**A)** and BrANCH (**B**), respectively), women underwent neuropsychological testing and blood draws to quantify NULISAseq plasma proteomics and orthogonal measures of AD biomarkers. Analyses evaluated associations of menopause signature scores and individual proteins with cognitive and AD outcomes, adjusting for age, education (for cognitive outcomes), and BMI (for AD pathology outcomes). **C.** In WRAP, higher menopause signature scores associated with poorer memory performance and higher levels of plasma p-tau217/Aβ42. In BrANCH, higher menopause signature scores associated with faster executive function decline and higher levels of plasma p-tau181/Aβ42. **D.** Individual menopause proteins CCL11, SNAP25, and p-tau231 showed most consistent associations with cognitive and AD outcomes across cohorts. **E.** CCL11 was the individual menopause protein that showed the most replicable associations, significantly relating to poorer memory outcomes in both aging cohorts.

In both aging cohorts, we evaluated associations of the menopause signature with cognitive and AD biomarker outcomes (Figs.5A–B). In WRAP, higher menopause proteomic signatures were associated with worse memory performance (β= −0.15, 95% CI= −0.26, −0.05, *p*=.005) and elevated plasma p-tau217/Aβ42 levels (β=0.090, 95% CI=0.006, 0.173, *p*=.04) (Fig.5C); the association with executive function was not significant (β=0.02, 95% CI= −0.08, 0.12, *p*=.70). In BrANCH, higher baseline menopause proteomic signatures were associated with faster executive function decline (i.e., β= −0.012, 95% CI= −0.026, 0.000, *p*=.05) and higher plasma p-tau181/Aβ42 (β=0.132, 95% CI=0.008, 0.256, *p*=.04) (Fig.5C); the association with memory decline were not significant (β= −0.006, 95% CI= −0.025, 0.012, *p*=.50).

In post-hoc analyses, CCL11 was the only marker among all menopause-related proteins to significantly relate to cognitive outcomes across both older adult cohorts (Figure 5D). Higher CCL11 related to worse cross-sectional memory (WRAP) and steeper longitudinal declines in memory and executive function (BrANCH) (Fig.5E). CCL11 also related to higher p-tau181/Aβ42 in BrANCH, but not p-tau217/Aβ42 in WRAP. As expected, p-tau231 significantly associated with plasma p-tau/Aβ42 ratios in both cohorts, as well as poorer memory and executive function performance in WRAP but not BrANCH. Of note, WRAP is specifically enriched for family history of AD, whereas BrANCH is a cohort of typical brain aging which may contribute to some differential relationships (e.g., memory/AD biomarkers versus executive functioning).

### Cross-platform validation cohort

#### Menopause proteomic shifts replicate in a large independent cohort.

**Figure 6.**
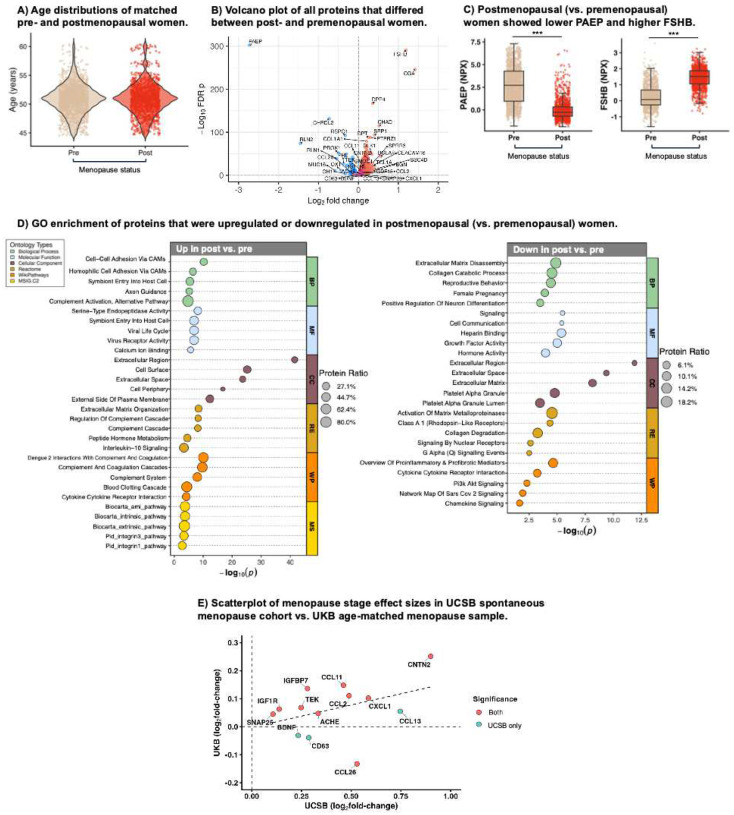
Menopause proteomic shifts replicate cross-platform in age-matched pre- and postmenopausal women in the UK Biobank. **A.** Pre- and postmenopausal women aged 45–60 were propensity-matched on age with a 1:1 ratio to form the analytic sample (*N*=2,814). **B**. Age-adjusted differential abundance analyses of Olink proteins demonstrated 1,146 upregulated and 154 downregulated proteins in postmenopausal (vs. premenopausal) women. **C**. Top hits included markers of ovarian hormone signaling, including progestogen-associated endometrial protein (PAEP) and FSH subunits beta (FSHB). **D**. Gene ontology (GO) enrichment analysis revealed menopause-associated downregulation of proteins enriched for growth factor and reproductive signaling and upregulation of proteins enriched for cytokine signaling, complement activation, and extracellular matrix catabolism. **E.** Effect sizes for menopause proteins identified in UCSB demonstrated concordance with those in UKB, showing strong overlap of the largest-magnitude hits in both cohorts. *p<.05, **p<.01, ***p<.001.

A total of 44% of plasma proteins (1,300 out of 2,923) were differentially abundant (FDR *p*<.05) between age-matched post- and premenopausal women (1,146 upregulated, 154 downregulated) from the UKB (Table S3, Fig.6B). Top hits included markers of ovarian hormone signaling, including decreased progestogen-associated endometrial protein (PAEP) and increased FSH subunits beta (FSHB) and alpha (CGA), supporting the biological relevance of our menopause classifications (Fig.6C). GO analysis revealed broader menopause-associated downregulation of proteins enriched for growth factor and reproductive signaling and upregulation of proteins enriched for cytokine signaling, complement activation, and extracellular vesicle–related processes (Fig.6D). Finally, we assessed whether the menopause proteins identified in the UCSB HAS cohort also showed menopause stage differences in the UKB sample. Of the 13 menopause proteins measured on both platforms, 9 clearly replicated across both cohorts. The effect sizes demonstrated concordance (r=0.39, *p*=.18) and strong overlap of the largest-magnitude hits in both datasets (Fig.6E).

### Sensitivity analyses

Sensitivity analyses are described in the Extended Data. In brief, findings were robust to (1) further restriction of the UCSB HAS sample by chronological age (i.e., 47–53; *N*=54), (2) exclusion of participants who self-reported a history of bilateral oophorectomy in aging cohorts (*N*=3 WRAP, *N*=19 BrANCH), (3) exclusion of p-tau231 from the menopause signature in aging cohorts, and (4) adjustment for potential confounds in the UKB sample.

## DISCUSSION

We used attamolar-sensitivity, multi-plex proteomics in a unique cohort of midlife women to identify a blood-based signature of menopause enriched for inflammatory, metabolic, synaptic, and AD biology. This signature tracked with hormones and did not associate with age. Across perimenopausal, early postmenopausal, and aging women, vasomotor symptoms related to exaggerated increases in pro-inflammatory proteins. The menopause proteomic signature was associated with worse cognitive outcomes and elevated levels of AD plasma biomarkers in two independent brain aging cohorts. Top menopause-related proteomic shifts robustly replicated across platforms in a large, independent cohort of age-matched pre- and postmenopausal women. Collectively, our multi-cohort design spanning five-plus decades of aging women highlights molecular signatures of menopause that overlap with proteomic drivers of later life brain health, underscoring menopause as a key biological transition that holds relevance to later life neurodegenerative risk.

The predominance of immune/inflammatory molecules in the menopause proteomic signature (i.e., CCL13, IL-12p70, CXCL1, CCL2, CCL11, TEK) alongside the upregulation of pathways related to cytokine signalling and the complement cascade align with prior findings of rises in systemic inflammation post-menopause – together supporting the theoretical conceptualization of menopause as an inflammatory event.^2,20^ The menopause signature was also enriched for proteins possibly involved in synaptic signaling (i.e., ACHE, SNAP-25, CNTN2), consistent with animal models of menopause demonstrating widespread disruptions in synaptic plasticity, spine density, and cholinergic signalling.^4,27,28^ We further observed increases in proteins implicated in AD (i.e., p-tau231, BACE1), which extends existing reports of increases in Aβ and tau-PET across the menopause transition.^12–14^ Of note, there were not significant differences in other tau epitopes (i.e., p-tau217, p-tau181) by menopause stage. Recent data suggest p-tau231 may represent the earliest biomarker of tau hyperphosphorylation^29^ which may more sensitively capture AD biologic changes in a midlife cohort. Concordant with this hypothesis, menopause-related increases in BACE1, which initiates formation of Aβ peptides and may be causally implicated in AD pathogenesis,^30^ may also reflect an acceleration in very early Aβ plaque biology, setting the stage for neurodegenerative risk in the decades to come. Although all women undergo menopause, most do not develop AD, and the clinical relevance of subthreshold elevations in p-tau231 and BACE1 in midlife women is currently unclear. Understanding how menopause and its sequelae modulate risk for AD biological changes in midlife and beyond is an urgently needed area of study.

Menopause proteomic differences tracked more strongly with hormones (i.e., estradiol, FSH) than age, suggesting that the observed biological changes may be primarily driven by ovarian versus chronological aging. We found that many proteomic shifts were most strongly linked to FSH, including key targets implicated in AD pathobiology and synaptic processes (i.e., BACE1, ACHE, CNTN2).^30–32^ FSH receptors are abundantly expressed in AD-vulnerable brain regions, and preclinical AD models show that FSH can trigger development of AD pathology, neuroinflammation, and cognitive deficits – effects that are reversed with FSH blockade.^33^ In the current study, lower estradiol was primarily linked to increases in pro-inflammatory markers (i.e., CCL26, CCL2, TEK), aligning with the established role of estrogens in modulating immune responses.^34^

We also observed that vasomotor symptoms of menopause were associated with menopause proteomic signatures, across both midlife and aging women. These associations were driven primarily by pro-inflammatory targets, with CCL2 (i.e., MCP-1) emerging as most replicable. Prior studies have reported that vasomotor symptom severity is associated with vascular inflammation^35,36^ (including CCL2^37^), plus cardiovascular risk and AD-related brain changes.^17,18^ Vasomotor symptoms may signal a less adaptive physiological response to menopause that confers risk for adverse brain aging and other chronic diseases. It is unclear why only night sweats (not hot flashes) were associated with the menopause signature in midlife women, and only hot flashes (not night sweats) were associated with the menopause signature in WRAP. However, given their shared etiology^38^, this inconsistency may relate to recall bias and imprecision introduced by self-report measures. There were also unexpected associations of vaginal dryness with lower (i.e., more premenopausal) menopause signature scores, the mechanistic significance of which is unclear.

Our study is among the first to demonstrate associations of menopause-related proteomic changes to later-life cognitive and brain outcomes. Across both aging cohorts, menopause signature scores were associated with worse cognitive outcomes and plasma p-tau/Aβ42. The most replicable target across cohorts was CCL11 (i.e., eotaxin-1), which consistently predicted poorer memory performance in older women. CCL11 is a chemokine strongly related to aging that has been implicated in a range of neuroinflammatory disorders, including AD.^39,40^ In animals, systemic CCL11 administration induces hippocampal microglial reactivity and impaired neurogenesis and can promote “brain aging” even in young animals.^41^ In the present study, increased CCL11 also tracked closely with FSH elevations, raising the possibility of FSH-induced neuroinflammation as a mechanistic link between menopause and cognitive aging.

Strengths of this study include the unique cohort with rigorously defined menopause stages to identify proteomic shifts of menopause which largely replicated in a cross-platform, age-matched validation cohort. These data provide compelling evidence that the observed proteomic changes were driven by endocrine rather than chronological aging. Of note, 3 of the targets identified in the UCSB cohort did not show concordant elevations in the UKB sample (i.e., BDNF, CD63, CCL26), which may reflect proteomic platform differences, spurious findings, or the coarse evaluation of menopause status in the UKB. Other limitations include that hormones were only measured at one timepoint despite known fluctuations, the reliance on self-reported menopause symptoms, and that protein measurements in blood reflect multiple tissue sources of expression. Longitudinal studies mapping the trajectories of hormones, multiomics, symptoms, and brain health across the menopause transition will be critical to disentangle the complex interplay between endocrine, neurologic, and chronological aging.

We defined a blood-based proteomic signature of spontaneous menopause, enriched for inflammatory, metabolic, synaptic, and AD biology. We demonstrated that inflammatory changes are especially pronounced in women with vasomotor symptoms, highlighting CCL2 as a signal of this physiologic susceptibility that may persist into later life. We further illustrated that menopause biology relates to markers of cognitive aging and AD risk. These findings shine new light on the biological processes connecting menopause to neurodegenerative risk, affirming the importance of menopause as a critical window for our foundational understanding of brain aging. There are currently no tools to monitor brain health during menopause. We identify blood levels of CCL11 and p-tau231 as potential biomarker candidates of menopause brain health which warrant further examination. Leveraging menopause to identify pathways that influence brain aging may inform midlife, person-specific dementia prevention approaches. Interventions to directly delay ovarian aging or promote adaptive physiologic responses to menopause also hold promise for preventing AD and fostering cognitive resilience in women.

## Supplementary Material

Supplementary Files

This is a list of supplementary files associated with this preprint. Click to download.

- extdataS14S19.xlsx
- extdataS1S13.xlsx
- spontaneousmenopauseproteomicsextdata.docx
